# The mediating effects of parenting style on the relationship between parental stress and behavioral problems in girls with precocious puberty in Korea: a cross-sectional study

**DOI:** 10.1186/s12887-023-04172-1

**Published:** 2023-07-12

**Authors:** Ahreum Kwon, Young Il Cho, Hyo Jin Kim, Junghwan Suh, Dong Hee Kim

**Affiliations:** 1 Department of Pediatrics, College of Medicine, Yonsei University, 50-1 Yonsei-ro, Seodaemun-gu, Seoul, Republic of Korea; 2 College of Police and Criminal Justice, Dongguk University, 30, Pildong-ro 1-gil, Jung-gu, Seoul, Republic of Korea; 3 College of Nursing, Seoul National University, 103, Daehak-ro, Jongno-gu, Seoul, Republic of Korea; 4 College of Nursing Science, Sungshin Women’s University, 55, Dobong-ro 76ga-gil, Gangbuk-gu, Seoul, Republic of Korea

**Keywords:** Parenting, Stress, Problem behavior, Precocious puberty

## Abstract

**Background:**

This study aimed to examine the mediating effects of parenting style on the relationship between parental stress and behavioral problems of girls with precocious puberty.

**Methods:**

This cross-sectional study analyzed a convenience sample of 200 mothers of girls with precocious puberty at a university hospital located in a metropolitan area. The Parental Stress measurement, Parents as Social Context Questionnaire, and Korean version Child Behavior Checklist (K-CBCL) 6–18 were measured via self-report questionnaires. Descriptive, t-test, Pearson correlation, and bootstrapping analyses were used to analyze the data.

**Results:**

Negative parenting styles had a full mediating effect on the relationship between parental stress and internalizing and externalizing behavioral problems.

**Conclusions:**

Care plans for parents of girls with precocious puberty should be designed and applied in health care settings to reduce internalizing and externalizing behavioral problems by decreasing negative parenting styles.

## Background

Precocious puberty refers to early signs of puberty that occur at an age less than 2.5 standard deviations below the average age of a population [1]. In Korea, precocious puberty is considered when its characteristics occur before the age of 8 in females, and 9 in males [2, 3]. Globally, sexual maturation is occurring more rapidly than in the past, and incidences of precocious puberty are increasing [4–6]. In Korea, the number of children with precocious puberty increased by about 1.5 times from 95,524 to 2017 to 166,645 in 2021 [7]. Girls are more affected than boys, with the female to male ratio being between 10:1 and 20:1 [8].

Precocious puberty involves physical problems such as early breast and genital development, accelerated physical growth and bone age, and short stature due to premature closure of growth plates. The onset of a disease or significant physical alteration at this stage can be mean added stress, generating more behavioral problems [9]. Hence, behavioral problems, along with physical symptoms, are recognized as major precocious puberty problems that require intervention [10].

Behavioral problems are often broadly classified as either externalizing or internalizing behavior. Externalizing behaviors are characterized by negative emotions directed against others, anger, aggression, disobedience, frustration, and underdeveloped self-regulation skills, leading to undercontrolled behaviors. In contrast, internalizing behaviors include withdrawal, fearfulness, inhibition, depression, and anxiety, and are characterized by negative emotions directed at oneself rather than others, along with rigid self-regulation and over-control [11].

Children with precocious puberty undergo physical, mental, psychological, and social changes much earlier than their peers. Rapid changes in physique and an early development of reproductive organs can lead to an imbalance in physical and mental maturation and create a negative body image [12, 13]. These rapid, unexpected changes may also trigger psychological problems—such as anxiety, depression, and stress—due to having a different body shape from one’s peer group [14–16]. Moreover, during the treatment for precocious puberty that is administered over several years, children can experience anxiety and fear related to the prognosis, treatment, and side effects [17]. In a recent study, 28% of girls with precocious puberty were diagnosed with psychiatric disorders, and the most prevalent comorbid condition was social anxiety disorder (13%) [18]. Also, they have problems that the social interactions may shrink, or negative relationships experience between peers due to physical and psychological problems [18]. Particularly, girls have a lower self-esteem [19] and lower body satisfaction compared to boys [20]. Girls and boys exhibit different patterns of behavioral problems [21], and girls comprise the major population in precocious puberty [4, 5]. Therefore, this study focused on the behavioral problems of girls.

Behavioral problems are thought to be a product of complex interactions between biological and environmental risk factors at the individual, family, and community levels [22]. In particular, parental factors have been suggested to play an important role in the risk for developing behavioral problems [23]. Thus, parental factors that directly or indirectly influence children’s behaviors should be assessed in an effort to inform and develop future intervention protocols.

The physical, psychological, and social problems experienced by girls with precocious puberty can become a source of stress for parents. Along with the stress of raising a child with a disorder, mothers are affected by their daughters’ physical and psychosocial changes and may feel worried, anxious, and guilty about the symptoms and prognosis of precocious puberty [10]. Moreover, monitoring and treatment for precocious puberty are expensive [24]. In fact, parents of children who go through a precocious puberty are reported to experience more parenting stress than parents of children who do not go through a precocious puberty [25, 26]. This parental stress, in turn, can contribute to a child’s behavioral problems [27, 28]. In addition, when parents experience such stress, their parenting style can be negatively affected [29, 30]. According to Abidin’s Parenting Stress Model [31], parenting stress experiences related to the role of being a parent, including characteristics of the child that are seen as stressors, will influence parenting style, which can have an impact on the child’s psychosocial adaptation. In this context, understanding the mediating effect of parenting style on the relationship between parental stress and girls’ behavioral problems will identify parameters that can explain the behavioral problems of girls with precocious puberty and suggest a direction for future intervention based on these parameters.

## Methods

### Aim

This study examines the mediating effects of parenting style on the relationship between parental stress and behavioral problems in girls with precocious puberty.

The following hypotheses will be tested:

#### Hypothesis 1

Parental stress has a direct effect on the behavioral problems (externalizing and internalizing behavioral problems) in girls with precocious puberty.

#### Hypothesis 2

Parenting style (positive and negative parenting styles) may have an indirect effect on the relationship between parental stress and behavioral problems in girls with precocious puberty.

### Study design and data collection procedure

This study used a cross-sectional design. After obtaining approval from the Severance Hospital Institutional Review Board (IRB) (4-2020-1077), data were collected with the cooperation of the hospital’s pediatric endocrinology outpatient clinic staff from March to September 2021. After approval, a notice was posted on the bulletin board of the children’s hospital for parents who visited the outpatient clinic. In order to prevent mothers from feeling obliged to participate, the study’s explanation and data collection were conducted by researchers unrelated to the direct clinical team. Mothers who refused to participate did not experience any coercion. Informed written consent was obtained from mothers accompanying the pediatric patients prior to inclusion in the study. Self-report survey questionnaires were provided to mothers to fill out in a separate space of the outpatient clinic, while the patients waited for regular check-ups with physicians. The time required to complete the questionnaire was approximately 30 min. After completing the questionnaire, each participant received a token of appreciation.

### Participants

The participants were selected from a pediatric clinic at a university-affiliated medical center located in metropolitan Seoul. The participants of this study included only girls because most of the cases reported were of girls diagnosed with precocious puberty. As mothers are the main childcare providers in Korean culture [32], fathers were excluded from this study. The inclusion criteria for participation were mothers who (1) had girls diagnosed with precocious puberty, (2) had girls over 6 years old, (3) had no health-related complaints at the most recent medical visit, (4) were able to understand and fill out the questionnaire, and (5) understood the purpose of this study and consented to participation. All mothers who met the inclusion criteria were invited to participate in the study, yielding a convenience sample. The number of participants was calculated as 184 using the G*Power 3.1.9.2 sample calculation program [33] with a significance level of 0.05, power of 95%, medium effect size of 0.15 for multiple regression, and 12 independent variables. Given the probability of an approximately 10% loss of participants and to ensure higher accuracy, at least 200 participants were considered. Of the initial 200 participants recruited for the study, all were included in the final analysis with no incomplete questionnaires.

### Measurements

#### General characteristics

The mothers’ age, gender, religion, perceived economic status, and level of education were assessed. The mothers’ menarche age, the girls’ physical development speed, and the children’s dependence score were measured using a 10-point numerical rating scale. The children’s age, height, weight, age diagnosed with precocious puberty, and Gonadotropin Releasing Hormone (GnRH) agonist treatment were checked.

#### Parental stress

To measure the level of stress in caring for a girl with precocious puberty, we used the parental stress measurement tools for mothers of children with chronic diseases developed by Kim [34] and modified and supplemented by Park [35]. This self-report questionnaire comprised 38 items on four dimensions including disease status and prognosis, disease management, family and personal roles, and interpersonal relationships. Each item was rated on a four-point Likert scale ranging from 1 (not worried at all) to 4 (very worried). The higher the score, the higher the stress level. Cronbach’s alpha was 0.96 in this study.

#### Parenting style

Parenting style was measured using the Korean version of the Parents as Social Context Questionnaire (K-PSCQ), validated by Jeong and Shin [36], based on the PSCQ developed by Skinner et al. [37]. This questionnaire comprised 23 items and 6 dimensions of parenting style (warmth, rejection, structure, chaos, autonomy, and coercion). Warmth, structure, and autonomy were positive dimensions, whereas rejection, chaos, and coercion were negative dimensions. The items were rated on a four-point Likert scale where 1 indicated “not at all true,” 2 “not very true,” 3 “sort of true,” and 4 “very true.” In the current study, Cronbach’s α values for positive parenting styles and negative parenting styles were 0.71 and 0.86, respectively.

#### Behavioral problems

The children’s behavioral problems were measured using the Korean version of the Child Behavior Checklist (K-CBCL) 6–18, developed by Achenbach and Rescorla [38] for parents to evaluate the behaviors of children aged 6 to 18, and translated and standardized by Oh and Kim [39] in Korea. The norm-referenced CBCL was completed by parents; it describes a child’s emotions and behaviors during the previous six months. The items measured specific behavioral problems on a three-point Likert scale where 0 indicated “not true,” 1 “somewhat or sometimes true,” and 2 “very true or often true.” Two subscales of the K-CBCL were used to measure child behavioral problems: (1) internalizing (anxious/depressed, withdrawn/depressed, and somatic complaints) and (2) externalizing problems (rule breaking behavior, aggressive behavior, social problems, thought problems, and attention problems). As suggested by Achenbach [40], a T-score of 64 or greater on a scale of total behavioral problems and a T-score of 70 or greater on a scale of each behavioral problem constitutes clinically significant symptoms. Cronbach’s α values for internalizing problems and externalizing problems were 0.81 and 0.78, respectively.

### Data analysis

We performed data analysis using PASW software (version 20.0) and the PROCESS macro (version 2). The percentages, means, and standard deviations (SDs) were calculated for the participants’ general characteristics. To capture variations in the scores of parental stress, parenting style, and child behavioral problems, we obtained the means, SDs, and possible ranges of scores. Pearson correlation coefficients were computed to identify the relationships between child behavioral problems and other variables, such as parental stress and parenting style. Reliability coefficients of the scales were examined by Cronbach’s alpha coefficients. Estimates in the mediational model of interest were calculated by employing the causal modeling process proposed by Baron and Kenny [41]. Significance of the indirect effects in the models was examined by a bootstrapping method. Specifically, 5,000 samples were generated to compute empirical distribution of the indirect effects, and a 95% confidence interval was calculated to test the significance of the indirect effects.

## Results

### Participants’ general characteristics

A total of 200 mothers were included, and their average age was 42.2 years. The average number of children was about two. Fifty-three (52.5%) mothers did not prescribe to any religion. Seventy-five mothers were college or university graduates. The average age of maternal menarche was 12.8 years. The average age of their children was 10 years, and the girls’ average Body Mass Index (BMI) was 19.2. Moreover, the mean age of diagnosis of precocious puberty was 8.3 years, and 92.5% of girls received Gonadotropin Releasing Hormone (GnRH) agonist treatment (Table 1).

**Table 1:** General characteristics of participants (N = 200)

	Characteristics	Categories	N (%)	M ± SD(Range)
Mother	Age (year)			42.20 ± 3.77(33 ~ 55)
	Number of children			1.93 ± 0.61(1 ~ 3)
	Religion	Yes	95(47.5)	
		No	105(52.5)	
	Education level	High school	21(10.5)	
		College or University	149(74.5)	
		Graduate school	30(15)	
	Age of Menarche (year)			12.77 ± 1.43(9 ~ 16)
Child	Age (year)			9.99 ± 1.33(6 ~ 13)
	BMI			19.22 ± 2.89 (11.65 ~ 27.77)
	Age at diagnosis			8.34 ± 1.56(3 ~ 13)
	GnRH agonist treatment	Yes No	185(92.5) 15(7.5)	

### Descriptive statistics and correlations among variables

Descriptive statistics for the major variables are shown in Table 2. The mean score of parental stress was 75.11 (SD = 24.8), whereas those of positive and negative parenting styles were 3.03 (SD = 0.41) and 1.88 (SD = 0.47), respectively. The mean score of total behavioral problems was 45.75 (SD = 9.25), internalizing problems was 46.64 (SD = 8.28), and externalizing problems was 46.60 (SD = 8.13); all of the above scores were calculated using the T-score.

**Table 2:** Parental stress, parenting style, behavioral problems scores(N = 200)

Variables	M ± SD	Min	Max	Range	
Parental stress	75.11 ± 24.80	38	151	38 ~ 152	
Positive parenting behaviors	3.03 ± 0.41	2.07	4	1 ~ 4	
Negative parenting behaviors	1.88 ± 0.47	1	3.10	1 ~ 4	
Total behavioral problems (T-score)	45.75 ± 9.25	30	67		
Internalizing problems	46.64 ± 8.28	37	76		
Externalizing problems	46.60 ± 8.13	37	73		

A statistically significant relationship was found between parental stress and negative parenting styles (r = .305, p < .001), internalizing behavioral problems (r = .378, p < .001) and externalizing behavioral problems (r = .203, p = .004). (Table 3).

**Table 3:** Correlation between major variables

	Parental stress r(*p*)	Positive parenting styles r(*p*)	Negative parenting styles r(*p*)	Total behavioral problems r(*p*)	Internalizing problems r(*p*)	Externalizing problems r(*p*)
Parental stress	1					
Positive parenting styles	0.064(0.371)	1				
Negative parenting styles	0.305(< 0.001)	− 0.149(0.035)	1			
Total behavioral problems	0.353(< 0.001)	− 0.040(0.570)	0.534(< 0.001)	1		
Internalizing problems	0.378(< 0.001)	− 0.038(0.595)	0.504(< 0.001)	0.822(< 0.001)	1	
Externalizing problems	0.203(0.004)	0.011(0.878)	0.495(< 0.001)	0.830(< 0.001)	0.603(< 0.001)	1

### Statistical analysis of indirect effects

**Fig. 1:**
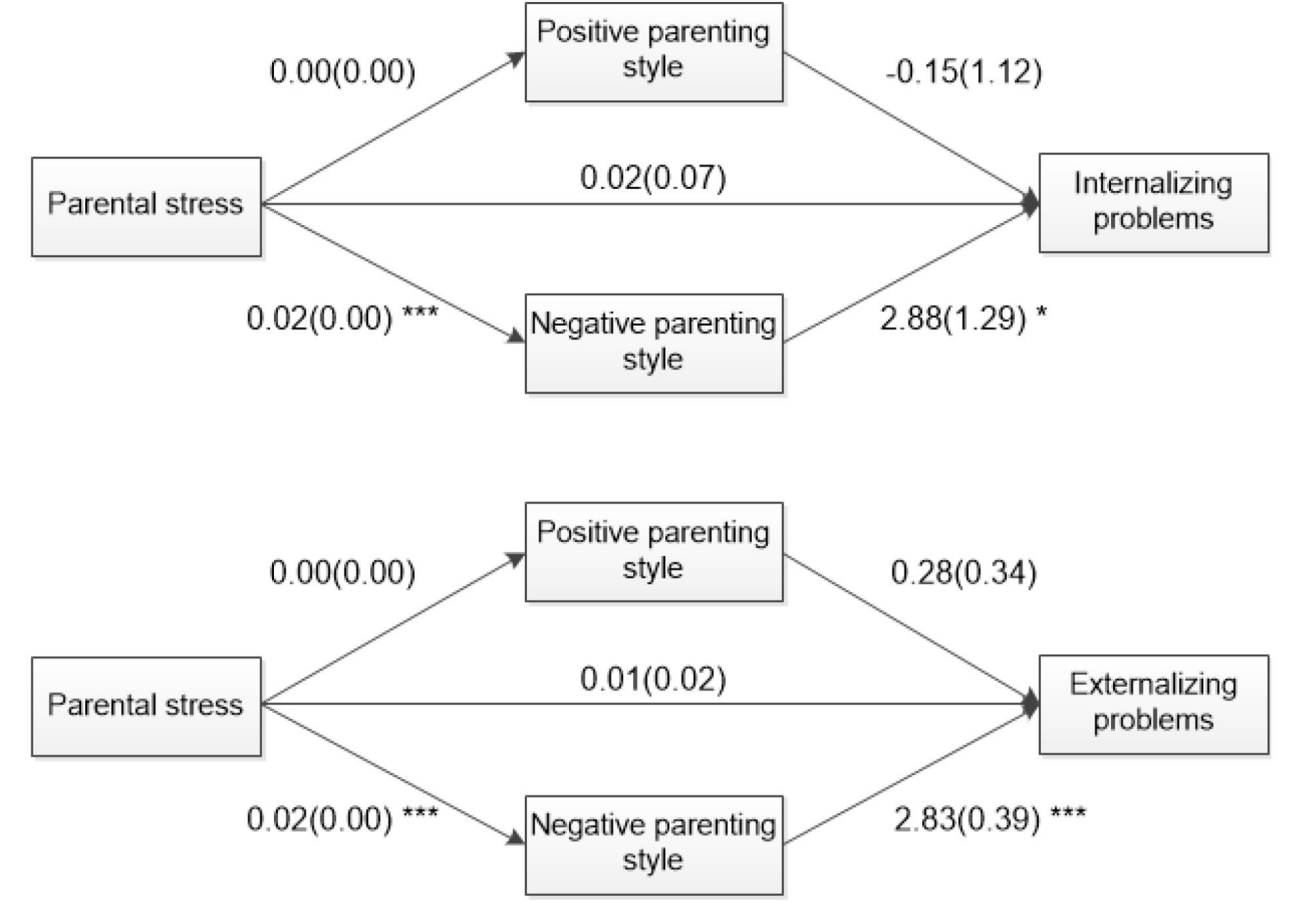
Mediation model of parenting styles on the child’s problems and unstandardized regression coefficient. * *p* < .05, ** *p* < .01, *** *p* < .001

To examine the hypotheses of this study, the indirect effects of the mother’s positive and negative caring behaviors on the relationship between parental stress and outcome variables were estimated and tested for their significance based on the bootstrapping results. The results for the outcome variables of internalizing and externalizing behaviors are reported in Table 4; Fig. 1.

**Table 4:** The mediating effects of positive and negative parenting styles on the relationship between parental stress and child’s internalizing/externalizing behavioral problems

Outcome Variables	Model 1 (X → Y)			Model 2 (X → M1 & M2)			Model 3 (X, M1, & M2 → Y)		
	Path	b (s.e.)		Path	b (s.e.)		Path	b (s.e.)	
Internalizing Behavior	Intercept	43.02(5.43) ^***^		Intercept	8.82(0.35) ^***^		Intercept	31.71(13.38) ^*^	
	X→Y	0.07(0.07)		X →M1	0.00(0.00)		X →Y	0.02(0.07)	
				Intercept	4.38(0.30) ^***^		M1→Y	-0.15(1.12)	
				X→M2	0.02(0.00) ^***^		M2→Y	2.88(1.29) ^*^	
Externalizing Behavior	Intercept	42.27(1.82) ^***^		Intercept	8.82(0.35) ^***^		Intercept	27.39(4.02) ^***^	
	X→Y	0.06(0.02) ^**^		X →M1	0.00(0.00)		X →Y	0.01(0.02)	
				Intercept	4.38(0.30) ^***^		M1→Y	0.28(0.34)	
				X→M2	0.02(0.00) ^***^		M2→Y	2.83(0.39) ^***^	

Note: X, M1, and M2 represent parental stress, positive parenting styles, and negative parenting styles, respectively. Y represents behavioral problems. ^*^p < .05, ^**^p < .01, ^***^p < .001.

Regarding the outcome variable of internalizing behaviors, the indirect effects of positive caring behaviors on the relationship between parental stress and internalizing behaviors were not significant, *est*. = -0.0006, 95% CI = [-0.0067, 0.0024]. By contrast, the indirect effects of negative caring behaviors on the relationship between parental stress and internalizing behaviors were significant, *est*. = 0.0485, 95% CI = [0.0240, 0.0816]. Specifically, parental stress increased mothers’ negative caring behaviors which, in turn, were positively associated with children’s internalizing behaviors. Additionally, the direct effects of parental stress on internalizing behaviors were not significant, *b* = 0.02, *SE* = 0.07, *p* = .83. Therefore, positive caring behaviors showed indirect effects in the model for the internalizing behaviors.

For the outcome variable of externalizing behaviors, the indirect effects of positive caring behaviors on the relationship between parental stress and externalizing behaviors were not significant, *est*. = 0.0012, 95% CI = [-0.0017, 0.0071]. By contrast, the indirect effects of negative caring behaviors on the relationship between parental stress and externalizing behaviors were significant, *est*. = 0.0477, 95% CI = [0.0256, 0.0729]. In particular, parental stress increased mothers’ negative caring behaviors, which were positively associated with the level of the child’s externalizing behaviors. Additionally, the direct effects of parental stress on externalizing behaviors were significant, *b* = 0.01, *SE* = 0.02, *p* = .53. Therefore, the negative caring behaviors yielded complete indirect effects in the model for the externalizing behaviors.

## Discussion

The final analysis of this study did not support our first hypothesis. In other words, we found that parental stress did not directly affect children’s behavioral problems. However, findings from this study partially supported our second hypothesis. This is because positive parenting styles did not show an indirect effect; only negative parenting styles showed an indirect effect on the child’s externalizing as well as internalizing behavioral problems. The results of hypotheses 1 and 2 suggest that negative parenting styles show a full mediating effect between parental stress and the child’s behavioral problems. In other words, stress increases the negative parenting styles in child-rearing situations, which may cause further problematic behaviors in children. Research shows that negative parenting style is an important variable that explains why children develop problematic behaviors when parents experience stress.

The higher the parental stress, the more skeptical the parents become of the parental role and the more likely they are to act excessively towards their children to solve the difficulties. Consequently, in the interaction between parents and children, parents lack warmth and acceptability, use inconsistent parenting styles, and have expectations that are not appropriate for the child’s development [42, 43]. In previous studies, the group with high parenting stress demonstrated a greater percentage of the neglectful permissive and controlling negative parenting styles [44]. These negative parenting styles affect children’s externalized or internalized behavioral problems. Rejection, chaos, coercion, and control are negative parenting styles that parents of chronically ill children may exhibit while caring for their children and managing the children’s symptoms [45]. These styles may negatively affect the children’s confidence, making them less confident in their behavior and adversely affecting their self-regulatory abilities [46]. For children with chronic disease, it is important that effective self-management can be achieved at an early age, enabling the management of their conditions throughout life [47]. However, children raised with negative parenting styles may not develop qualities such as autonomy and advocacy, lowering their self-management behavior [48, 49].

Accordingly, evidence shows that negative parenting styles are risk and mediation factors that can cause internalized and externalized behavioral problems in children. In addition, certain vulnerable groups of parents, such as those with children experiencing precocious puberty, are more susceptible to the effects of parental stress and, therefore, may be more likely to exhibit negative parenting styles. In light of this situation, educational programs and other interventions are warranted to lower the parents’ stress, minimize negative parenting styles, and promote parenting styles that aid children’s development.

Notably, our results revealed that positive parenting styles did not mediate behavioral problems. This result differs from the previous studies’ finding that positive parenting styles act as a protective factor and can positively affect children’s behavioral problems [50, 51]. However, our findings that negative parenting styles act as risk factors for behavioral problems in children are consistent with previous studies [52–54]. The results on the effects of positive and negative parenting styles on behavioral problems are inconsistent. We suggest that a variety of theoretical and methodological studies should be conducted to thoroughly analyze the protective and risk factors affecting children’s problem behaviors as well as to identify effective intervention factors so that a beneficial intervention plan for children and parents may be developed and implemented.

Lastly, unlike individualistic cultures that emphasize individuality and independence, Korea adopts a relational culture [55], particularly through a unique parenting style wherein parents view their children not as separate individuals but as an extension of themselves as a whole [56]. In a study conducted in Korea, it was found that parents felt guilt as they believed that their parenting was directly related to their child’s diagnosis [57], and role stress was the most influential factor for parent’s quality of life [24]. Considering the cultural context of parent-child relationships, parental factors such as the stress and parenting style that occur between parents and children, are variables that should be considered more important for children’s behavioral problems in Korea.

Our study has several limitations. First, potential bias may be present due to unmeasured variables that affect behavioral problems in children with precocious puberty, either directly or indirectly, such as height, temperament, personality, and family stress, which are ubiquitous problems in most observational studies [58, 59]. Second, the behavioral problems were measured according to the parents’ reports only. Direct responses on behavioral problems from the children themselves need to be included in future studies, which may be helpful to confirm the consistency between reports from children and parents. Third, this study only included girls with precocious puberty and mothers, who are traditionally the main childcare providers in the Korean culture. The inclusion of boys and fathers in future studies will be useful to explain other aspects of the relationship between behavioral problems, parental stress, and parenting styles in children with precocious puberty. Fourth, the participants were recruited from a single hospital in Korea, possibly limiting the ability to generalize our findings.

Despite these limitations, this study revealed that negative parenting styles are the key variable mediating the parental stress and the behavioral problems among girls with precocious puberty. In the majority of cases, treatment for precocious puberty is based on symptom management [60]. In addition, family interventions are currently limited to the provision of information relating to the disease and education pertaining to medication [61]. However, we propose that interventions related to parental factors need to be developed in health care settings, including parenting styles as well as symptom management or disease information educations. We submit that intervention strategies for parents that include enhancing parent-child communication skills, improving problem-solving skills, developing of more effective parenting skills, and nurturing the ability to deal with the negative emotions experienced during parenting will help reduce behavioral problems in children [62, 63].

## Conclusion

Examining the relationships among parental variables, such as parental stress and parenting styles on the behavioral problems of girls with precocious puberty, we found moderating effects of negative parenting style on the relationship between parental stress and internalizing and externalizing behavioral problems of girls with precocious puberty. Therefore, the development and application of interventions may help parents reduce their negative parenting style, which was a major variable mediating children’s behavioral problems in this study. Moreover, these efforts will help alleviate children’s internalizing and externalizing behavioral problems, thus enhancing the quality of life of both children and parents.

## Data Availability

The data is not publicly available because it contains information that could compromise the privacy and consent of research participants, but is available to the corresponding author on reasonable request.
